# Polymer-to-Carbon Conversion: From Nature to Technology

**DOI:** 10.3390/ma12050774

**Published:** 2019-03-06

**Authors:** Swati Sharma

**Affiliations:** Karlsruhe Institute of Technology, Institute of Microstructure Technology, Hermann-von-Helmholtz-Platz-1, 76334 Eggenstein-Leopoldshafen, Germany; swati.sharma@kit.edu; Tel.: +49-721-608-29317

**Keywords:** polymer-derived carbon, glassy carbon, pyrolysis, microdevices, nanomanufacturing

## Abstract

Glassy carbon is derived from synthetic organic polymers that undergo the process of coking during their pyrolysis. Polymer-to-carbon conversion (hereafter referred to as PolyCar) also takes place in nature, and is indeed responsible for the formation of various naturally occurring carbon allotropes. In the last few decades the PolyCar concept has been utilized in technological applications, i.e., specific polymers are patterned into the desired shapes and intentionally converted into carbon by a controlled heat-treatment. Device fabrication using glassy carbon is an excellent example of the use of the PolyCar process in technology, which has rapidly progressed from conventional to micro- and nanomanufacturing. While the technique itself is simple, one must have a good understanding of the carbonization mechanism of the polymer, which in turn determines whether or not the resulting material will be glassy carbon. Publications that comprise this special issue shed light on several aspects of the formation, properties and performance of glassy carbon in the cutting-edge technological applications. The results of detailed material characterization pertaining to two important research areas, namely neural electrodes and precision glass molding, are presented as examples. I hope that the readers will enjoy as well as benefit from this collection.

## 1. Introduction

Conversion of natural polymers into carbon under extreme temperature and pressure is responsible for the formation of large deposits of graphite, coal, petroleum and diamonds at different depths below the surface of the Earth [1]. These carbon materials originate from various natural polymers and other hydrocarbons, such as those present in plant and biological matter. The conditions for their formation may range from sudden and massive tectonic movements to an underground burial for thousands of years causing an extremely slow disintegration of the material under pressure. Natural precursor polymers often also contain an appreciable fraction of non-carbon atoms that contributes to the structure of the ensuing carbon. Consequently, various carbon materials in the Earth’s mantle exhibit significant differences in terms of microstructure, properties and physical states. Even within one type of natural elemental carbon, for example coal, variations exist [2].

The fundamental difference between the aforementioned carbon forms and glassy carbon is the fact that glassy carbon does not occur naturally. First reports on the preparation of this material are less than 100 years old [3]. This was the time when the potential of carbon in modern technology was recognized, and had created the need for an artificial and relatively pure form of carbon in large quantities. Most of the polymers used for making glassy carbon are therefore the synthetic ones, with known chemical structures. A controlled annealing process ensures the release of all non-carbon atoms in an orderly manner, hence, unlike natural carbon forms glassy carbon features a high purity. The deciding factor on whether or not a polymer will yield glassy carbon is its carbonization mechanism, more specifically, if the material goes through a semi-solid phase during its pyrolysis. This is known as the coking process [4]. Some pertinent definitions such as coking and charring, polymer-to-carbon conversion (PolyCar) process, and a classification of polymers based on their carbonization mechanism is provided in the review article [4] published in this special issue. In addition, a description of PolyCar technique and PolyCar-compatible polymers that yield glassy carbon is provided. Finally, some recently fabricated miniaturized glassy carbon structures are listed as representative examples.

There are two research articles published in this collection that detail the influence of the preparation and processing methods on the performance of glassy carbon structures used in (i) neural implants and (ii) glass molding tools. The research article by Vomero et al. [5] is focused on thin-film glassy carbon microelectrodes obtained by the PolyCar technique, where the precursor polymer is a common photoresist, SU-8. On the other hand, Grunwald et al. [6] utilize commercially available glassy carbon, which is subject to grinding and polishing prior to its use as a precision glass molding tool. Interestingly, electrodes and high-temperature molds are two of the oldest applications of this material. Nonetheless, the use of electrodes has advanced from larger solid-state batteries to miniaturized biomedical devices, and the molds for shaping glass have become progressively smaller due to the optical elements required for the cutting-edge laser technology.

Vomero et al. [5] evaluated the conformability of glassy carbon thin-film electrodes employed for electrocortiography. Glassy carbon is cytocompatible and chemically inert, and is therefore a favorable material for biomedical devices. However, an important additional feature required in the case of interactive implants is their conformability, i.e., how well do the electrode structures adapt the shape of the host organ or tissue. The authors presented a detailed investigation of the structural shrinkage caused by the PolyCar process and carried out a design optimization for the resulting glassy carbon structures in order to determine their conformability, and thus usability in neural implants. The team also reported on the effect of electrochemical pretreatment processes used for the surface activation in view of neural interactions, which can be useful for various other biomedical applications as well. The fact that glassy carbon can be obtained from common photo-patternable polymers renders it suitable for the fabrication of biomedical aids such as functional implants, scaffolds, and other 2- and 3D structures that entail cytocompatibility, electrical conductivity as well as electrochemical and mechanical stability.

The second research article, contributed by Grunwald et al. [6], presents a detailed description of the correlation between surface finishing methods and tool wear during high-temperature precision glass (fused silica) molding. The glassy carbon molding tools used in this study are milli- to centimeter scale. The authors conducted a rigorous characterization of the subsurface damage and the defect generation patterns in the material after grinding and polishing at different parameters, and confirmed that the surface finishing strategies do induce wear and roughness in the molds. Fabrication of molds for shaping the materials with a high melting point or processing temperature is another popular application of glassy carbon. In fact, the large-scale production of the early camera lenses was based on glassy carbon molds [7], which revolutionized the camera industry. This application has become even more pertinent in today’s technology due to the increasing need for optical elements at a miniaturized scale. Molding entails a thermally stable and atomically smooth surface. Glassy carbon features a low thermal expansion coefficient [4], exhibits thermal shock resistance, and can withstand reasonably high mechanical loads. Moreover, as the size of the optical elements decreases to submicron scales, glassy carbon can also be obtained by carbonization of pre-patterned polymers in a batch-fabrication [8].

Evidently, while neural implants benefit from glassy carbon’s electrochemical stability [9], chemical inertness and biocompatibility, the glass molding tools exploit its thermal and mechanical properties. The optimization processes and effect of material preparation parameters on their intended application presented in both articles is expected to be extremely useful in determining the manufacturing parameters. Further details and the methodologies used by the authors of the aforementioned articles can be accessed in the respective, open access articles of this special issue.

Over the years, the PolyCar process has emerged as a strong technological tool that can be used in combination with advanced polymer patterning techniques such as photolithography. This process has also been used for carbon fiber preparation for over five decades. Custom-designed glassy carbon objects, such as crucibles, substrates and vessels can also be procured commercially. Based on these facts, it is reasonable to assume that manufacturing with glassy carbon using the PolyCar process is commercially viable. Given the current trends in the development of advanced polymers and their patterning techniques, it is expected that PolyCar and glassy carbon will play a more significant role in the next-generation devices.

This collection was an effort to bring together the knowledge of the chemistry of the carbonization process, classification of polymer-derived carbon, and some representative examples of glassy carbon structures and devices. I believe that the published articles fulfil this purpose, and hope that they receive attention from carbon- as well as micro/nano manufacturing communities. I thank all contributing authors for submitting their work to this special issue.
